# MicroRNA expression profiling in human acute organophosphorus poisoning and functional analysis of dysregulated miRNAs

**DOI:** 10.4314/ahs.v18i2.18

**Published:** 2018-06

**Authors:** Haijun Yuan, Mei Yuan, Yonghong Tang, Biao Wang, Xiangyang Zhan

**Affiliations:** 1 The Second Affiliated Hospital, University Of South China, Department of Emergency; 2 The second affiliated Hospital, University Of South China, Department of Neurology

**Keywords:** MicroRNA, expression profiles, human, acute organophosphorus pesticide poisoning, signaling pathways

## Abstract

**Objective:**

Acute organophosphorus(OP) pesticide poisoning is associated with dysfunctions in multiple organs, especially skeletal muscles, the nervous system and the heart. However, little is known about the specific microRNA (miRNA) changes that control the pathophysiological processes of acute OP poisoning damage. We aimed to explore miRNA expression profiles and gain insight into molecular mechanisms of OP toxic effects.

**Methods:**

MicroRNA expression was analyzed by TaqMan Human MicroRNA Array analysis and subsequent validated with quantitive PCR. The targets of the significantly different miRNAs were predicted with miRNA prediction databases, and pathway analysis of the predicted target genes was performed using bioinformatics resources.

**Results:**

37 miRNAs were significantly different in the sera of poisoned patients compared to the healthy controls, including 29 miRNAs that were up-regulated and 8 miRNAs that were down-regulated. Functional analysis indicated that many pathways potentially regulated by these miRNAs are involved in skeletal muscle, nervous system and heart disorders.

**Conclusion:**

This study mapped changes in the serum miRNA expression profiles of poisoning patients and predicted functional links between miRNAs and their target genes in the regulation of acute OP poisoning. Our findings are an important resource for further understanding the role of these miRNAs in the regulation of OP-induced injury.

## Introduction

Acute OP poisoning is a threat to human health worldwide and causes significant morbidity and mortality, especially in developing countries. Patients exposed to these pesticides suffer from several health problems and deaths, mainly cholinergic system dysfunction, neurological abnormalities, paralysis, muscle damage and cardiac abnormalities. However, further investigations are needed for understanding the pathophysiology of the main target organ of OP, and enable the development of new therapies to reduce the mortality rate.

MicroRNAs (miRNAs) are small, non-protein coding RNA chains that are approximately 22 nucleotides in length. miRNAs modulate most cellular functions, including cell differentiation, proliferation, migration, development, metabolism and apoptosis.^1^,^2^ Accordingly, small RNAs are critical regulators of normal development, physiology, pathology and organ damage and repair. Therefore, we deduced that miRNAs may play a crucial role in the pathophysiological processes of acute OP poisoning.Recently, it is believed that the alterations in the miRNA expression profile mediate toxicity from toxicant.^3^

We hypothesised that circulating miRNA signatures may be used as a novel tool to study OP-induced injury. In the current study, we used a TaqMan Human MicroRNA Array from Applied Biosystems to analyse the miRNA expression profiles of serum samples obtained from acute OP poisoning patients compared to normal, healthy participants. 8 differentially expressed miRNAs were selected for further validation using qRT-PCR. Then, we performed a preliminary analysis of the Kyoto Encyclopedia of Genes and Genomes (KEGG) pathway for the potential target genes of the most differentially expressed miRNAs. Our research provides information on the miRNA expression in the human acute OP poisoning.

## Materials and methods

### Clinical subjects

This study protocol was approved by the Human Ethics Committees Review Board at the Second Affiliated Hospital of the University of South China (Hengyang, China), and written informed consent was obtained from all of the participants prior to their enrollment.

A total of 34 individuals who were admitted to the emergency department of the Second Affiliated Hospital of the University of South China with intentional oral OP(-methamidophos) from October 2012 through September 2014 were selected and enrolled sequentially into the study as patient subjects (poisoning group). Their diagnosis was based on a history of oral consumption of organophosphorus pesticide methamidophos, the presence of characteristic symptoms of pesticide poisoning, and reduced blood acetylcholinesterase (AChE) activity. The durations of the pesticide methamidophos ingestion to blood sample collection (at admission) were 3 hours to 24 hours. The doses of the ingested 50% methamidophos were from 20 ml to 40 ml. The blood AchE activity of patients was decreased by 70% or more. The control group consisted of 30 healthy volunteer individuals recruited from the Health Screening Center of the same hospital. The poisoning and control groups were matched by age and gender, and were from the same area, and lived and worked under comparable circumstances, and there was no past history of exposure to toxic substances. Potential subjects who were diagnosed with diabetes mellitus, hypertension, hyperlipidaemia, inflammatory diseases, or malignant tumours during the clinical and laboratory examinations were excluded from this study.

### Blood sampling

A peripheral venous blood sample (5 ml) was collected from each patient in the poisoning group at admission, and fasting venous blood samples were collected from healthy control subjects in the morning. The blood samples were centrifuged at 3000 r/min for 5 min, and then the sera were collected and saved at −20°C, and used for miRNA analysis.

### Total RNA extraction from subject samples

Total RNA was extracted from each serum sample using the TRIzol reagent (Invitrogen, Carlsbad, CA, USA) and purified using an Ambion mirVana miRNA Isolation Kit (Ambion, USA). This procedure efficiently recovered all RNA molecules, including miRNAs. The RNA quality and quantity were measured using a NanoDrop ND-1000 spectrophotometer (NanoDrop Technologies, Wilmington, DE, USA), and the RNA integrity was determined by gel electrophoresis.

### TaqMan low-density array and RT-qPCR analysis

For microRNA array analysis, 3 randomly case samples and 3 randomly normal samples were selected. TaqMan Human MicroRNA A and B Arrays, version 3.0 (Applied Biosystems, Foster City, CA, USA) were used to screen the samples for the expression of 754 serum miRNAs on an ABI Prism 7900 HT Fast RealTime PCR system. To increase the sensitivity of the TaqMan Low Density Array, pre-amplification was performed after reverse transcribing the total RNA. The reverse transcription was performed using 500 ng of total RNA, Megaplex™ RT Primers (Applied Biosystems, USA) and the TaqMan MicroRNA Reverse Transcription Kit (Applied Biosystems, USA) to synthesise cDNA. The reaction mixtures were incubated at 16°C for 2 min, at 42°C for 1 min, and at 85°C for 5 min, and then they were cooled at 4°C. Real-time PCR was performed using TaqMan Universal PCR Master Mix II, No UNG (Applied Biosystems, USA) and a TaqMan Human MicroRNA Array Set, also from Applied Biosystems. The real-time PCR cycles consisted of the UNG enzyme reaction at 50°C for 2 min and a pre-denaturation cycle of 94.5°C for 10 min, followed by 40 cycles of 97°C for 30 sec and 59.7°C for 1 min. All reactions were performed as specified in the manufacturer's protocol. The miRNA expression was calculated based on the cycle threshold (Ct) values and normalised to U6 snRNA and RNU44. The assays were completed in triplicate. Amplification signals were computed with 7500 software v2.0.6 (Applied Biosystems, USA). The relative miRNA expression levels were calculated by the 2^-ΔΔCt^ method.

### Selection of miRNA candidates

After the TaqMan low-density array and RT-qPCR analysis, the expression of an individual miRNA was defined as being significantly different from the compared groups if the P-value was < 0.05 and the fold change was more than 2.

### Validation of expressed miRNAs by qRT-PCR analysis

To validate the miRNA array results in our study, four randomly up-regulated miRNAs (miR-29b, miR-133a, miR-214 and miR-590) and four down-regulated miRNAs(miR-125b, miR-141-5p, miR-150 and miR-642a) were selected for further qRT-PCR confirmation with all samples of poisoning group and control group. The methodological details of qRT-PCR and internal control (cel-miR-39) were as previously described.^4^ The miRNAs were reversely transcribed into cDNAs by using the ReverTra Ace qPCR RT Kit (TOYOBO, Japan). The qPCR then conducted using the SYBR Premix Ex TaqTM kit (TakaRa, Dalian, China). qRT-PCR was processed in 96-well plates on a 7500HT analyzer (Applied Biosystems, USA) according to the direction of the reagents in triplicate. The sequence-specific RT primers and the qPCR primers are shown in Table 1, which was provided by GenePharma (Shanghai, China). The threshold cycle (Ct) values used in the calculation were the means of the triplicates. The relative expression of each miRNA was normalized using the Ct obtained from internal control using the 2^−ΔΔCt^ method.

**Table 1:** qRT-PCR primer sequences of 8 miRNAs

miRNA	Primer sequences
miR-29b	RT primer: 5′-GTCGTATCCAGTGCAGGGTCCGAGGTATTCGCACTGGATACGACAACACTG-3′
	Forward primer: 5′-GGCGAGCTAGCACCATTTGAAAT-3′
miR-133a	RT primer: 5′-GTCGTATCCAGTGCAGGGTCCGAGGTATTCGCACTGGATACGACCAGCTG-3′
	Forward primer: 5′-AGAGGATTTGGTCCCCTTCAAC-3′
miR-214	RT primer: 5′-GTCGTATCCAGTGCAGGGTCCGAGGTATTCGCACTGGATACGACACTGCCT-3′
	Forward primer: 5′-TTTGTTGACAGCAGGCACAGAC-3′
miR-590	RT primer: 5′-GTCGTATCCAGTGCAGGGTCCGAGGTATTCGCACTGGATACGACCTGCACT-3′
	Forward primer: 5′-CGGCGGCGAGCTTATTCATAAA-3′
miR-125b	RT primer: 5′-GTCGTATCCAGTGCAGGGTCCGAGGTATTCGCACTGGATACGACTCACTTG-3′
	Forward primer: 5′- AGTAGGTGTTCCCTGAGACCCTAA-3′
miR-141–5p	RT primer: 5′-GTCGTATCCAGTGCAGGGTCCGAGGTATTCGCACTGGATACGACCCATCTT-3′
	Forward primer: 5′-GGCCACGATAACACTGTCTGGTA-3′
miR-150	RT primer: 5′-GTCGTATCCAGTGCAGGGTCCGAGGTATTCGCACTGGATACGACCACTGG-3′
	Forward primer: 5′-GAGGTAGGATCTCCCAACCCTTGTA-3′
miR-642a	RT primer: 5′-GTCGTATCCAGTGCAGGGTCCGAGGTATTCGCACTGGATACGACCAAGAC-3′
	Forward primer: 5′-GAGAGTAGGTCCCTCTCCAAATGT-3′
All miRNAs	Common reverse primer: 5′- TCCAGTGCAGGGTCCGAGGT-3′

### miRNA target gene prediction and pathway analysis

The identification of targets of miRNAs is crucial for elucidating their function. However, the miRNA-target interaction is very complicated. At present, several target prediction algorithms have been developed, but they show poor overlap in their outputs, suggesting that they generate many false-positive and false-negative predictions. 5 Here, putative target genes of miRNAs were predicted using two miRNA databases: TargetScan (http://www.targetscan.org/) and miRDB (http://mirdb.org/miRDB/index.html). We compared the predicted target genes from both algorithms and selected the predicted targets that were identified by both databases. We conducted the analyses separately for the up-regulated and down-regulated miRNAs.

For pathway analysis of the predicted targets, the KEGG pathway analysis, which is a systematic analysis of gene functions in terms of the networks of genes and molecules, was conducted on the predicted target genes to identify their associated signalling pathways. KEGG pathway analysis was carried out using DAVID Bioinformatics Resources 6.7 (http://david.abcc.ncifcrf.gov/).

### Statistical analysis

Statistical testing was conducted with the assistance of the SPSS 13.0 software. An independent-samples t-test was performed to determine which miRNAs were modulated at a significant level (P < 0.05). The P value was adjusted by Bonferroni correction to counteract the problem of multiple testing.

## Results

### miRNA expression

The TaqMan Human MicroRNA Array showed 232 miRNAs that were expressed in the sera of the patients and the healthy controls at different levels. Compared to the normal samples, 37 miRNAs were significantly altered in the sera of the patients, including 29 miRNAs that were up-regulated and 8 miRNAs that were down-regulated (Table 2).

**Table 2:** Differential expression of 37 microRNAs in the sera of AOPP patients and healthy controls

Name	Fold change	P-value	Regulation	Name	Fold change	P-value	Regulation
miR-133a	75.889	0.032	Up	miR-561	3.128	0.010	Up
miR-599	31.833	0.004	Up	miR-1267	3.127	0.006	Up
miR-135a	10.237	0.009	Up	miR-590-5p	3.075	0.038	Up
miR-545	8.989	0.018	Up	miR-375	2.857	0.047	Up
miR-661	7.852	0.000	Up	miR-1290	2.719	0.004	Up
miR-138	6.709	0.019	Up	miR-454	2.697	0.046	Up
miR-623	6.359	0.015	Up	miR-625-3p	2.611	0.037	Up
miR-515-3p	5.639	0.001	Up	miR-126	2.573	0.005	Up
miR-590-3p	5.328	0.048	Up	miR-28-5p	2.011	0.008	Up
miR-362	5.010	0.000	Up	miR-27a-5p	2.005	0.000	Up
miR-194	4.485	0.022	Up	miR-155	−2.006	0.001	Down
miR-1291	4.265	0.000	Up	miR-122	−2.110	0.000	Down
miR-29b	4.146	0.000	Up	miR-193b	−2.321	0.000	Down
let-7g	4.087	0.000	Up	miR-642a	−2.342	0.000	Down
miR-142-3p	4.068	0.027	Up	miR-141-5p	−2.849	0.001	Down
miR-192	3.821	0.033	Up	miR-125b	−3.215	0.001	Down
miR-223	3.625	0.000	Up	miR-301	−4.065	0.005	Down
miR-483-5p	3.563	0.007	Up	miR-150	−5.319	0.006	Down
miR-214	3.519	0.011	Up				

**Figure 1:**
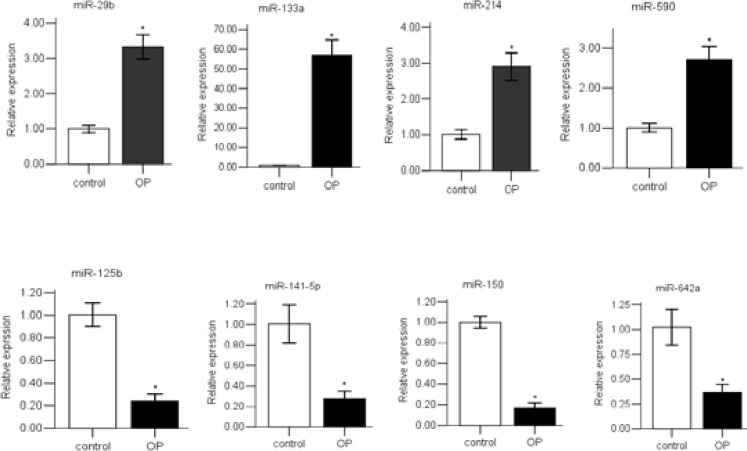
The expression of selected miRNAs were validated by qRT-PCR.

miRNAs that were differentially expressed in the AOPP group and the control group. Candidate miRNAs exhibited a greater than 2-fold difference, P < 0.05. miRNAs described as “up” are higher in the AOPP patients than in the healthy controls, whereas “down” indicates lower expression in the AOPP group than in the control group. To verify the accuracy of array results, 8 randomly miRNAs (miR-29b, miR-133a, miR-214, miR-590, miR-125b, miR-141-5p, miR-150 and miR-642a) were selected for further qRT-PCR analysis with all patients and controls. The results were comparable with the array results (Figure 1).

Compared to the control group(control), the expression of miR-29b, miR-133a, miR-214, and miR-590 in poisoning group(OP) were significantly higher, while the expressions miR-125b, miR-141-5p, miR-150, and miR-642a in poisioning group(OP) were significantly lower (*P <0.05).

### KEGG pathways associated with the differentially expressed target genes

The target genes of the differentially expressed miRNAs were predicted. In total, 2290 and 374 target genes were up-regulated and down-regulated by miRNAs, respectively. To understand the functions of the two types of genes, we utilised KEGG pathway analysis with DAVID Bioinformatics Resources. The major component of KEGG is the PATHWAY database that consists of graphical diagrams of biochemical pathways, including most of the known metabolic pathways and some of the known regulatory pathways. The KEGG enrichment analysis pathways, which contained many of the significantly differentially expressed target genes (P < 0.05), were identified in the up-regulated and down-regulated miRNA groups, respectively. In total, 29 KEGG pathways were overrepresented among the target genes in the up-regulated miRNA group, and they are shown in Table 3. Among these pathways are “axon guidance”, “ErbB signalling pathway”, and “long-term potentiation”, etc. Moreover, two KEGG pathways were significantly represented among the target genes in the down-regulated miRNAs group, “mTOR signalling pathway” and “Ubiquitin mediated proteolysis”.

**Table 3:** Overrepresented KEGG pathways among the target genes of the up-regulated miRNAs

KEGG pathway Term	Number of Genes
hsa04360:Axon guidance	36
hsa04012:ErbB pathway	27
hsa04720:Long-term potentiation	22
hsa05214:Glioma	20
hsa05200:Pathways in cancer	64
hsa04722:Neurotrophin signalling pathway	31
hsa04810:Regulation of actin cytoskeleton	46
hsa05218:Melanoma	21
hsa04910:Insulin signalling pathway	32
hsa04010:MAPK signalling pathway	53
hsa04310:Wnt signalling pathway	34
hsa04350:TGF-beta signalling pathway	23
hsa04510:Focal adhesion	41
hsa05220:Chronic myeloid leukaemia	20
hsa04144:Endocytosis	37
hsa04916:Melanogenesis	23
hsa05215:Prostate cancer	21
hsa05210:Colorectal cancer	20
hsa04912:GnRH signalling pathway	22
hsa04730:Long-term depression	17
hsa04540:Gap junction	20
hsa04120:Ubiquitin-mediated proteolysis	27
hsa04530:Tight junction	26
hsa05211:Renal cell carcinoma	16
hsa04520:Adherens junction	17
hsa04020:Calcium signalling pathway	32
hsa05414:Dilated cardiomyopathy	19
hsa04130:SNARE interactions in vesicular transport	10
hsa04512:ECM-receptor interaction	17

## Discussion

Acute OP poisoning has became a major public health problem with more than 300,000 deaths each year around the world,^6^ most commonly due to organophosphorus, and China has one of the highest morbidity and mortality rates from OP poisoning. It is also known to cause suicide, homicide, and non-intentional and occupational poisonings. In China, as in other developing countries that lack surveillance systems, regulation, enforcement, training, access to information systems, and well-maintained personal protective equipment and that have large agriculturally based populations, the incidence is very high. Acute OP poisoning can cause serious health problems, especially neuropathy, muscular damage and cardiac dysfunction. However, the mechanisms for many of these pathophysiologies are unclear.

miRNAs have been widely studied in recent years because they play a critical role in the pathogenesis of diverse diseases. miRNAs are remarkably stable and can be readily quantified in human and animal sera and plasma. More importantly, circulating miRNAs demonstrate significant dynamic change under some pathological conditions and can partially reflect tissue damage.^7^,^8^ Circulating miRNAs play an important role in the pathogenesis of many diseases.

Recently, a study of zebrafish indicates that 14 miRNAs were differentially expressed ascribable to the treatments with fipronil, triazophos and their mixture.^9^ However, a role for miRNA in human acute OP poisoning has not been reported, and little is known about the miRNA networks that control pathophysiological processes in the patients. In this study, we used a genome-wide TaqMan low-density array technology to determine the serum miRNA expression levels in three randomly patients and three randomly healthy controls to identify biomarkers of tissue damage and to improve our understanding of this disease. Then we verified the expressions of miRNAs using qRT-PCR with all patients and controls. Interestingly, we also successfully identified a set of differentially expressed miRNAs between the poisoning patients and the healthy controls. Overall, by TaqMan low-density array analysis, 37 miRNAs were markedly altered in the sera of patients compared to the healthy controls, including 29 miRNAs that were up-regulated and 8 miRNAs that were down-regulated. Then, 4 up-regulated and 4 down-regulated miRNAs were selected for verification. We found results of qRT-PCR and array analysis correlated strongly. Thus, it appears that poisoning with methamidophos causes specific changes in human serum miRNA. Theexpression profiling may be useful for in-depth research into OP-induced injury. To date, no reports are available on the effect of organophosphorus poisoning on these miRNAs of the.

Organophosphorus compounds exert acute systemic toxicity through phosphorylation by inhibiting the enzyme acetylcholinesterase, thus causing acute neurotoxicity. Nervous system damage is the most common toxic effect of acute OP poisoning.^10^ The dysregulation of miRNAs may contribute to OP-induced neuropathies. We conducted a systematic literature review of studies on peripheral neuropathy-related miRNAs and found many studies on miRNAs and nervous system damage diseases. For example, recently, a few studies have indicated that in the pathogenesis of nervous system diseases, miR-29b,^11^ miR-138,^12^ and miR-155^13^ play an important role. Schwann cells are the principle glia of the peripheral nervous system (PNS). They function to support neurons, and many neuropathies involve Schwann cells. A recent study of sciatic nerve injury in mice indicated that a cohort of 22 miRNAs coordinates Schwann cell differentiation and de-differentiation through combinatorial modulation of their positive and negative gene regulators. miR-138 and miR-709 show the highest likelihood of regulating Egr2, Sox-2 and c-Jun expression following injury.^12^ Furthermore, Verrier et al. showed that miR-29a binds and inhibits PMP22 reporter expression through a specific miRNA seed-binding region.^11^ Peripheral myelin protein 22 (PMP22) is a dose-sensitive, disease-associated protein primarily expressed in myelinating Schwann cells. The over-expression of miR-29a enhances the association of PMP22 RNA with Argonaut 2, a protein involved in miRNA function, and it reduces the steady-state levels of PMP22, which plays an important role in Schwann cell injury. In contrast to miR-29a, miR-29b expression was significantly increased in the OP poisoning patients; in fact, both miR-29a and miR-29b belong to the same miR-29 family.

Another disease in which these miRNAs have been found is Guillain-Barré syndrome (GBS), which is an acute polyneuropathy disorder affecting the peripheral nervous system. Wang et al. showed that miR-155 is down-regulated in peripheral blood mononuclear cells (PBMCs) from GBS patients and that silencing miR-155 profoundly promotes the production of Th1-type cytokines in vitro.^13^ Interestingly, delayed onset distal polyneuropathy is a consequence of severe intoxication due to the inhibition of the neuropathy target esterase (NTE) enzyme,^14^ also known as patatin-like phospholipase domain-containing 6 (PNPLA6), in nervous tissues by certain organophosphorous compounds. Interestingly, we found that the PNPLA6 gene was targeted by miR-214 and miR-454 from the TargetScan database, and both miRNAs were up-regulated in our study. Whether or not miR-214 and miR-454 participate in the formation of epigenetic silencing complexes with PNPLA6 mRNA to induce-post-transcriptional gene silencing has not been reported; however, these data suggest that miR-214 and miR-454 may control the translation of PNPLA6 to contribute to delayed polyneuropathy. The significant changes in these miRNAs demonstrate that they may be novel biomarkers for OP-induced nervous system damage.

The paralysis of muscles and muscular damage are common in OP poisoning patients. Many studies have reported that deregulated miR-29b,15 miR-125b,12 miR-133a,^16^–^18^ miR-135,^19^ miR-155,^18^ miR-193b,^12^ miR-22318,^19^ and miR-36218 were connected with the pathogenesis of muscular diseases. Kornfeld et al. showed increased miR-29b transcription in the pectoral muscle of torpid bats.^15^ Endo et al. reported that miR-133a is a muscle-related miRNA, and deregulated miR-133a plays central regulatory roles in muscle disease.^16^ Interestingly, another study noted up-regulated miR-133a in the skeletal muscle of mdx mice, an animal model for human muscular dystrophy.^17^ Furthermore, in facioscapulohumeral muscular dystrophy (FSHD), another hereditary neuromusculardisorder, 29 miRNAs were found to be differentially expressed between FSHD and normal myoblasts, including miR-133a, miR-223 and miR-362, which were up-regulated, and miR-155, which was down-regulated.^18^ In fact, miR-133a is the most profoundly different miRNA on our list, and it may be one of the most important miRNAs in acute OP poisoning. Additionally, a degenerative miRNA, miR-135a, and an inflammatory miRNA, miR-223, are involved in the pathological pathways activated in skeletal muscle damage and regeneration by both dystrophin absence and acute ischaemia in MDX mice and in DMD patients.^19^ Furthermore, in Myotonic Dystrophy Type-2 (DM2), an autosomal dominant disease that mainly affects skeletal muscles, the heart and the central nervous system, many miRNAs are deregulated, and miR-125b and miR-193b are decreased.^12^ Therefore, altered miRNAs are responsible for muscle diseases. Interestingly, we observed that many days after their poisoning, many acute OP poisoning patients showed varying degrees of muscular weakness and atrophy. These symptoms are similar to those of the aforementioned muscular diseases, but currently there is no reasonable explanation for this similarity. We speculate that OP-induced muscular damage is related to these dysregulated miRNAs.

Cardiac complications accompany poisoning with organophosphates are extremely common, these complications may be serious and are often fatal and include electrocardiographic abnormalities, conduction defects, cardiac arrhythmias, cardiac troponin T (cTnT) and myocardial enzyme elevation, and myocardial infarction and failure, a series of complications that is attracting substantial attention. Nevertheless, the pathogenesis of cardiac toxicity from these compounds is not yet clearly defined. Careful electrocardiographic and enzymatic monitoring and cardiac complication treatment are necessary for organophosphate poisoning in all patients. Heart damage and electrocardiographic abnormalities are connected with many miRNAs, such as up-regulated miR-29b,^20^ miR-126,^21^ miR-133a,^22^ miR-142-3p^23^, miR-192^24^, miR-194^24^, and miR-590-5p^25^ and down-regulated miR-122^26^, miR-125b^26^ and miR-155^27^. All of these studies suggest that OP may impair cardiac health by altering miRNA expression. However, precisely how altered expression of these miRNAs affects cardiac complications needs clarification.

To investigate the function and intracellular signalling pathways of the two types of miRNAs in acute OP poisoning, we predicted the target genes of different miRNAs using two miRNA prediction databases (TargetScan and miRDB ), and we generated lists of 2290 and 374 potential gene targets of up-regulated miRNAs and down-regulated miRNAs, respectively. Then, we analysed the target genes with the KEGG pathway database, and we found that 29 KEGG pathways were overrepresented among the target genes in the up-regulated miRNA group, mainly axon guidance, long-term potentiation, the neurotrophin signalling pathway, the insulin signalling pathway, SNARE interactions in vesicular transport, the regulation of the actin cytoskeleton, the calcium signalling pathway, dilated cardiomyopathy, endocytosis, focal adhesion, gap junctions, tight junctions, adherens junctions, the ErbB signalling pathway, the MAPK signalling pathway, the Wnt signalling pathway, the TGF-beta signalling pathway, ECM-receptor interaction and ubiquitin mediated proteolysis (Table 3). The target genes of down-regulated miRNAs were involved in the mTOR signalling pathway and the ubiquitin mediated proteolysis. To our surprise, our target prediction analysis also revealed that miRNAs identified in our experiment and their target genes are associated with a number of pathophysiological pathways that could indirectly influence multiple organ dysfunctions, especially muscular, nervous and heart disorders. Knowing that these miRNAs might play an important role in OP-induced damage.

## Conclusion

Although there is no evidence presented for a causal relationship between one or more specific miRNAs and any of the adverse effects of methamidophos, our study lays the groundwork for further molecular studies to identify the novel deregulated miRNAs potentially involved in OP toxicity, and it provides insight into the pathways that are damaged in acute OP poisoning. These data will be central to understanding how complications develop and the molecular mechanisms of OP-induced injury. However, to understand how these dysregulated miRNAs and their targets interact with acute OP poisoning, further sample neural, muscular, heart, and liver biopsies from their cohorts to examine the mRNA profling and miRNA changes directly in the affected tissues are required. Manipulating the expression of these miRNAs may promote understanding of the underlying mechanisms that are critical for OP-induced damage and ultimately provide therapeutic benefits for monitoring, preventing and treating OP poisoning complications and concomitant pathological lesions.
